# Associations between serum lipid levels and the macular retinal volumes in patients with diabetes

**DOI:** 10.1371/journal.pone.0325500

**Published:** 2025-06-04

**Authors:** Yasuaki Mushiga, Norihiro Nagai, Yoko Ozawa

**Affiliations:** 1 Department of Ophthalmology, St. Luke’s International Hospital, Tokyo, Japan; 2 Department of Ophthalmology, Keio University School of Medicine, Tokyo, Japan; 3 Department of Clinical Regenerative Medicine, Fujita Medical Innovation Center Tokyo, and Eye Center, Fujita Health University, Haneda Clinic, Tokyo, Japan; Sanmenxia Central Hospital, Henan University of Science and Technilogy, CHINA

## Abstract

**Purpose:**

Macular retinal volumes can be measured by optical coherence tomography (OCT). However, the underlying pathogenesis was obscure. We compared the OCT and serum lipid data in participants with or without diabetes mellitus (DM) and diabetic retinopathy (DR) to explore the interpretation of the OCT data.

**Methods:**

Data for eye examinations and blood tests in 41 eyes of 41 participants (23 men; mean age 49.1 ± 8.3) were analyzed. Eyes without macular lesions were included.

**Results:**

Mean macular retinal volumes of ganglion cell layer (GCL) (P = 0.023) and neural retinal layers (NRL) including layers from internal to external limiting membranes (P = 0.013) were smaller in the DM without DR group than in the control group. Mean serum malondialdehyde-modified low-density lipoprotein (MDA-LDL) levels were higher in the DM without DR (P = 0.046) and with DR (P = 0.021) groups than in the control group. Serum low-density lipoprotein cholesterol (LDLC) levels showed a negative correlation with GCL volume (P = 0.005), and trends of negative correlations with retinal nerve fiber layer (RNFL) (P = 0.060) and NRL volumes (P = 0.051) in the control group. However, in the DM with DR group, LDLC levels showed significant positive correlations with RNFL (P = 0.002), GCL (P = 0.034), and NRL (P = 0.002) volumes. The DR group also showed positive correlations between MDA-LDL levels and RNFL (P < 0.001) and NRL (P = 0.006) volumes.

**Conclusions:**

Macular retinal volumes may decrease owing to DM and elevated serum lipid levels. However, the volume may increase as serum lipid levels elevate after DR development. Further studies are warranted to understand the pathogenesis.

## Introduction

Recent progresses in medical science and technology have enabled us to prevent blindness by treating severe diabetic retinopathy (DR) using surgical and medical methods [1]. Patients focus on achieving a better quality of vision as well as preventing substantial visual loss. Thus, retinal imaging techniques, such as optical coherence tomography (OCT), and visual acuity exams, are used to evaluate retinal neural changes in daily clinical practice. However, even if OCT detects subtle changes, the interpretation of these changes based on their underlying pathogenesis is not fully understood.

DR has long been recognized as a microvasculopathy [2–4], which exhibits the breakdown of blood-retinal barriers (BRBs). In particular, the inner BRB, which is composed of vascular endothelial cells of inner retinal capillaries that regulate the transport of serum substances to the retina [5,6], is a key component of DR pathogenesis, such as diabetic macular edema [7,8]. On the other hand, diabetes is currently viewed as a disease of the neurovascular unit where cellular interactions at the molecular levels may accelerate pathogenesis before minimal vascular impairments are visible [9]; diabetic retinal neural impairments can be observed before DR is diagnosed [10–14]. Next interest would be whether the serum substances may affect the retinal conditions in the absence of visible macular edema.

The impact of controlling blood sugar levels on preventing DR progression has been known, and recent interest has focused on managing dyslipidemia [15,16]. The Japanese clinical guidelines for DR suggest that appropriate management of dyslipidemia may prevent DR progression (https://www.nichigan.or.jp/Portals/0/resources/member/guideline/diabetic_retinopathy.pdf). Given that the retina is constantly exposed to light, which causes oxidative stress [17], abnormal lipid metabolism may generate advanced lipoxidation end products (ALEs), which form cross-links on tissue proteins and accumulate during aging and chronic diseases [18]. Clinical data have shown that abnormal lipid metabolism accelerates the progression of microangiopathy, most likely through the increased secretion of vascular endothelial growth factor (VEGF) [19]. However, the potential relationships between serum lipid levels and retinal neural tissue remain obscure.

In this cross-sectional study, we aimed to analyze healthy controls, patients with diabetes mellitus (DM) without diagnosed DR, and those diagnosed with DR to assess the relationship between retinal tissue volume at the macula, where visual acuity is determined, and serum lipid levels. All participants had a best-corrected visual acuity (BCVA) better than 0.8 in the decimal scores and 0.10 in the logarithm of the minimum angle of the resolution (logMAR), and no history of macular edema; thus, macular function was considered preserved. In contrast to previous data which showed influence of diabetic macular edema [20] or retinal atrophic changes [21] on retinal volumes, the current study showed the possible influence of tissue modification related to lipid metabolism on retinal volume in diabetes. The study explored DR pathogenesis, providing cautions for data interpretation in the presence of a diabetic background, which would be of value in both daily clinical practice and future clinical studies.

## Materials and methods

### Ethics statement

This study adhered to the tenets of the Declaration of Helsinki, was approved by the Ethics Committee of St. Luke’s International University (approval number: 20-R058) and was registered as UMIN000040444. The cross-sectional observational study was performed from October 1, 2020 to September 30, 2022. All participants provided written informed consent.

### Participants

Twenty-nine patients with DM (15 with DR and 14 without DR) and 12 healthy age-matched participants were included. The inclusion criteria were BCVA better than 0.8 in decimal score (logMAR 0.10); axial length <27 mm. The eyes with macular diseases, including macular edema or epiretinal membrane with or without a history of treatment, and glaucoma were excluded. Data from the right eye were analyzed.

### Ocular examinations

All patients underwent BCVA measurements using refraction testing, slit-lamp examination, and binocular indirect ophthalmoscopy after pupil dilation with 0.5% tropicamide. A diagnosis of DR was determined by fundus examinations.

### OCT

Macular retinal volume was measured using a spectral-domain OCT system (Spectralis OCT; Heidelberg Engineering GmbH, Dossenheim, Germany) and the built-in device software. The three-dimensional OCT images were obtained by recording 97 scan lines in the 20x20 degree using automatic real time mean system (signal averaging system) of 4 times measurements at each line, by the well-trained certified orthoptists, and the quality of the images was confirmed by the retina specialists (NN and YO). The 3 mm diameter areas of the retinal nerve fiber layer (RNFL), ganglion cell layer (GCL), and neural retinal layers (NRL), including layers from internal to external limiting membrane, which was originally named as inner retinal layers by the manufacture, were measured automatically after confirming the segmentation lines by the retina specialists (NN and YO).

### Blood tests

Blood samples were obtained at the same day as the OCT recording. The blood tests were performed at SRL Inc. (Tokyo, Japan). Hemoglobin A1c (HbA1c) was measured by enzymatic assay, total cholesterol (TC) was with a cholesterol oxidase-peroxidase-voltammetry system, low-density lipoprotein cholesterol (LDLC), malondialdehyde-modified LDL (MDA-LDL), and high-density lipoprotein cholesterol (HDLC) were through enzyme-linked immunosorbent assay.

### Statistical analysis

All results were expressed as mean ± standard deviation. All statistical analyses were performed using commercially available software (SPSS version 29.0, IBM Corp., Armonk, NY, USA). The Kruskal–Wallis test, Mann–Whitney U test, Pearson’s chi-square test, and Pearson’s correlation coefficient were used for analyses. Statistical significance was set at P < 0.05.

## Results

Of the 41 eyes of 41 participants (23 men, mean age 49.1 ± 8.3), 12 eyes of 12 participants belonged to healthy individuals without any ocular diseases except for mild cataracts (control group), 14 eyes of 14 patients with DM had no DR (DM without DR group), and 15 eyes of 15 patients with DM had DR (DM with DR group) (Table 1). No significant differences were observed in age among the three groups (P=0.165). Although the BCVA was different among the three groups (P<0.001), all eyes had a better BCVA than 0.10 in logMAR.

**Table 1:** Characteristic of the eyes.

	Control	DM without DR	DM with DR	P^a^	P^b^	P^c^	P^d^
n	12	14	15				
Age (years)	44.8 ± 12.1 (27.0 to 61.0)	49.3 ± 5.3 (37.0 to 58.0)	52.4 ± 5.1 (42.0 to 60.0)	0.165	0.527	0.126	0.123
Gender (male), n (%)	2 (17)	9 (64)	12 (80)	0.003^**^	0.014^*^	0.001^**^	0.344
HbA1c (%)	5.1 ± 0.3 (4.7 to 5.5)	7.6 ± 2.3 (5.5 to 14.5)	8.3 ± 2.0 (6.0 to 12.6)	<0.001^***^	<0.001^***^	<0.001^***^	<0.001^***^
BCVA (logMAR)	−0.17 ± 0.03 (−0.18 to −0.08)	−0.12 ± 0.05 (−0.18 to −0.08)	−0.07 ± 0.08 (−0.18 to 0.10)	<0.001^***^	0.036^*^	<0.001^***^	0.123
RNFL (mm^3^)	1.00 ± 0.11 (0.77 to 1.14)	0.93 ± 0.11 (0.77 to 1.14)	1.14 ± 0.53 (0.74 to 2.94)	0.248	0.106	0.683	0.270
GCL (mm^3^)	1.13 ± 0.08 (1.00 to 1.22)	1.06 ± 0.07 (0.99 to 1.21)	1.06 ± 0.12 (0.87 to 1.35)	0.065	0.023^*^	0.067	0.880
NRL (mm^3^)	6.58 ± 0.35 (5.93 to 7.20)	6.25 ± 0.20 (5.94 to 6.60)	6.75 ± 1.21 (5.88 to 10.32)	0.123	0.013*	0.300	0.983

Data were shown by mean ± standard deviation (range).

^a^Kruskal–Wallis test for comparing 3 groups, ^b,c,d^Mann-Whitney test for comparing 2 groups; comparison of

^b^control and DM without DR,

^c^control and DM with DR, and

^d^DM without DR and DM with DR. Chi-square test for analyses of gender. DM, diabetes mellitus; DR, diabetic retinopathy; BCVA, best- corrected visual acuity; HbA1c, hemoglobin A1c; RNFL, retinal nerve finer layer; GCL, ganglion cell layer; NRL, neural retinal layers.

* P < 0.05,

** P < 0.01,

*** P < 0.001.

The mean GCL volume was significantly smaller in the DM without DR group than in the control group (P=0.023), and a similar trend was observed between the DM with DR and control groups (P=0.067). The mean NRL volume was significantly lower in the DM without DR group than in the control group (P=0.013).

Blood test data showed significant differences in MDA-LDL (P=0.048) and HDLC (P=0.001) among the three groups (Table 2). The mean MDA-LDL levels were significantly higher in the DM without DR (P=0.046) and DM with DR (P=0.021) groups than in the control group. In contrast, HDLC levels were lower in the DM without DR (P=0.004) and DM with DR (P<0.001) groups than in the control group.

**Table 2:** Blood test data.

Blood test	Control	DM without DR	DM with DR	P^a^	P^b^	P^c^	P^d^
TC (mg/μL)	211.3 ± 34.0 (160–259)	203.6 ± 53.9 (118–293)	205.5 ± 62.8 (133–386)	0.551	0.667	0.217	0.747
LDLC (mg/μL)	125.6 ± 32.1 (75–189)	119.9 ± 52.6 (40–215)	114.1 ± 33 (64–200)	0.733	0.595	0.456	0.847
MDA-LDL (mg/μL)	72.6 ± 21.0 (45–113)	109.5 ± 50.5 (55–226)	122.9 ± 87.2 (54–393)	0.048^*^	0.046^*^	0.021^*^	0.780
HDLC (mg/μL)	74.7 ± 18.1 (51–98)	53.3 ± 12.9 (36–77)	50.9 ± 13.9 (39–93)	0.001^**^	0.004^**^	<0.001^***^	0.561

Data were shown by mean ± standard deviation, range.

^a^Kruskal–Wallis test for comparing 3 groups, ^b,c,d^Mann-Whitney test for comparing 2 groups; comparison of

^b^control and DM without DR,

^c^control and DM with DR, and

^d^DM without DR and DM with DR;TC, total cholesterol; LDLC, low-density lipoprotein cholesterol; MDA-LDL, malondialdehyde-modified LDL; HDLC, high-density lipoprotein cholesterol.

* P < 0.05,

** P < 0.01,

*** P < 0.001.

**Fig 1:**
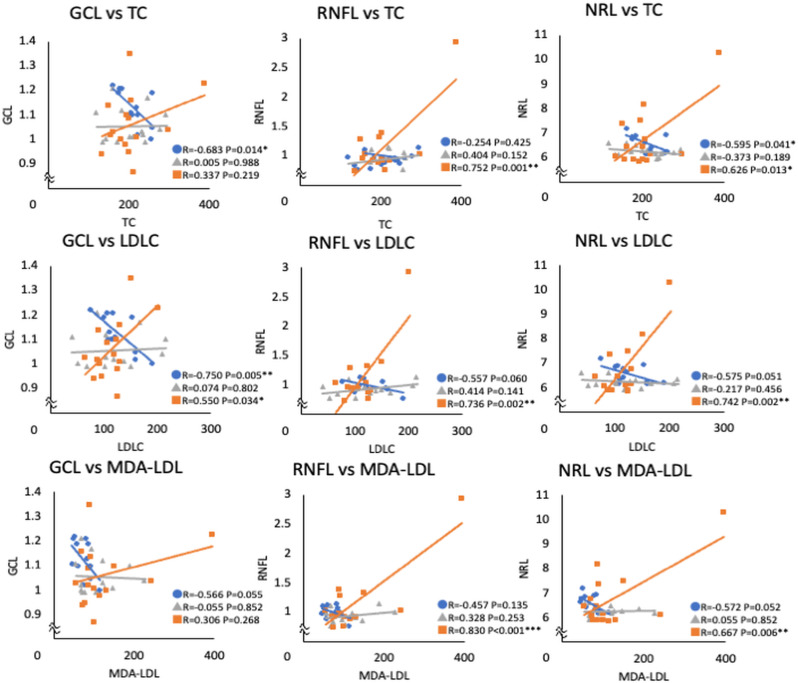
Scattered plots of serum lipid levels and the macular volumes of each layer. Pearson’s correlation coefficient. The correlation coefficient values showed opposite signs between the control and DM with DR groups. DM, diabetes mellitus; DR, diabetic retinopathy; TC, total cholesterol; LDLC, low-density lipoprotein cholesterol; MDA-LDL, malondialdehyde-modified LDL; HDLC, high-density lipoprotein cholesterol; RNFL, retinal nerve finer layer; GCL, ganglion cell layer; NRL, neural retinal layers. *P < 0.05, **P < 0.01, ***P < 0.001.

The associations between blood cholesterol levels and macular volumes in each layer were further investigated (Table 3, Fig 1). TC levels were negatively correlated with GCL (P=0.014) and NRL (P=0.041) volumes in the control group; however, they were positively correlated with RNFL (P=0.001) and NRL (P=0.013) volumes in the DM with DR group. Moreover, LDLC levels showed a negative correlation with GCL volume (P=0.005), and a similar trend with RNFL (P=0.060) and NRL (P=0.051) in the control group. However, in the DM with DR group, they showed positive correlations with RNFL (P=0.002), GCL (P=0.034), and NRL (P=0.002) volumes. Although MDA-LDL levels showed trends of negative correlations with GCL (P=0.055) and NRL (P=0.052) volumes in the control group, they showed positive correlations with RNFL (P<0.001) and NRL (P=0.006) volumes in the DM with DR group.

**Table 3:** Correlations between blood cholesterol levels and macular volumes.

		Control		DM without DR		DM with DR	
Blood Cholesterol Levels	Macular volume	R	P	R	P	R	P
TC	RNFL	−0.254	0.425	0.404	0.152	0.752	0.001^**^
	GCL	−0.683	0.014^*^	0.005	0.988	0.337	0.219
	NRL	−0.595	0.041^*^	−0.373	0.189	0.626	0.013^*^
LDLC	RNFL	−0.557	0.060	0.414	0.141	0.736	0.002^**^
	GCL	−0.750	0.005^**^	0.074	0.802	0.550	0.034^*^
	NRL	−0.575	0.051	−0.217	0.456	0.742	0.002^**^
MDA-LDL	RNFL	−0.457	0.135	0.328	0.253	0.83	<0.001^***^
	GCL	−0.566	0.055	−0.055	0.852	0.306	0.268
	NRL	−0.572	0.052	0.055	0.852	0.677	0.006^**^
HDLC	RNFL	0.521	0.083	−0.011	0.971	−0.28	0.311
	GCL	0.061	0.852	0.046	0.875	−0.282	0.308
	NRL	−0.078	0.81	−0.349	0.222	−0.311	0.259

Pearson’s Correlation Coefficient. DM, diabetes mellitus; DR, diabetic retinopathy; RNFL, retinal nerve finer layer; GCL, ganglion cell layer; NRL, neural retinal layers; TC, total cholesterol; LDLC, low-density lipoprotein cholesterol; MDA-LDL, malondialdehyde-modified LDL; HDLC, high-density lipoprotein cholesterol.

* P < 0.05,

** P < 0.01,

*** P < 0.001.

## Discussion

The mean macular GCL and NRL volumes were significantly smaller in the DM without DR group than in the control group. In blood tests, MDA-LDL levels were higher, and HDLC levels were lower in the DM with and without DR groups than in the control group. Serum TC levels showed negative correlations with GCL and NRL volumes in the control group, however, the TC levels showed positive correlations with RNFL and NRL volumes in the DM with DR group. Moreover, serum LDLC levels showed negative correlation with GCL volume, and showed trends of negative correlations with RNFL and NRL volumes in the control group, however, the LDLC levels showed positive correlations with RNFL, GCL, and NRL volumes in the DM with DR group. Serum MDA-LDL levels were also positively correlated with RNFL and NRL volumes in the DM with DR group.

In the current study, the mean GCL volume was lower in the DM without DR group than in the control group. This was consistent with a previous report by Spaide showing that thinning of the GCL was significant in patients with non-proliferative DR (PDR) compared with controls [21]. Supported data were also shown by Esmaeelpour et al. [22], Chen et al. [23], and Salvi et al. [24] who have shown significant thinning of the RNFL, which is composed of axons of retinal ganglion cells (RGCs), in the DM without DR group compared with the control group.

These findings suggested that RGCs were reduced in diabetic eyes from a relatively early phase, which was consistent with the results of diabetic animal studies. Sasaki et al. [13] and Zerbini et al. [25,26] demonstrated significant RGC loss and thinning of the RNFL/GCL in streptozotocin (STZ)-induced type 1 diabetic mice and in Akita mice carrying an insulin gene mutation, respectively. None of the models exhibited severe retinal vascular proliferation; however, neurodegeneration was evident. Sasaki et al. also showed that neurodegeneration was caused by oxidative stress and that the antioxidant, lutein, was effective in preventing neural changes [13]. In STZ-induced type 1 diabetic mice, a reduction in the synaptic protein synaptophysin has been shown by Kurihara et al., together with functional impairment in the inner retinal layer through angiotensin II type 1 receptor signaling [14]. Moreover, the functional decline in the retinal inner layers detected by oscillatory potentials in electroretinograms precedes visible fundus diabetic changes detected by ophthalmoscopy in patients with DM [10,11]. Animal studies [13,14] and human data [10,11] both showed that the inner retinal function was already impaired in the early diabetic process.

The current study confirmed that DM affects RGCs, and this influence was detected as a reduced retinal neural volume, prior to the clinical diagnosis of DR.

The mean MDA-LDL levels were significantly higher in the DM with and without DR groups than in the controls. MDA-LDL is an oxidized LDL produced by the oxidative modification of LDLC in vascular endothelial cells and contributes to the accumulation of lipids in the arterial wall. MDA-LDL is used as a marker for oxidative stress [27] and a risk factor for DR progression [28]. In general, MDA-LDL is taken up by and activates macrophages, which promote vascular and neural damage and disease progression, including DM [29]. Thus, an increase in MDA-LDL levels may accelerate inflammation in various tissues in DM.

Li et al. conducted a systematic review of previous studies on the relationship between DR progression and serum lipids, indicating elevated serum levels of LDLC, TG, and TC in patients with DR [30]. Moreover, Mondal et al. reported that LDLC levels were associated with intraocular VEGF levels [19]. Similarly, Zhang et al. reported that LDLC and TC levels were associated with intraocular VEGF levels [31]. Thus, serum LDLC may be involved in the vascular pathogenesis of DR. LDLC levels were not significantly different with or without DM in the current study. However, this finding could be attributed to the fact that LDLC may have been converted to MDA-LDL in the presence of DM, given that DM causes oxidative stress. [13].

The Wisconsin Epidemiologic Study of Diabetic Retinopathy showed that higher HDLC levels were associated with a lower risk for PDR [32]. HDLC plays a role in scavenging excess cholesterol and shuttling it to the liver; thus, maintaining homeostasis in cholesterol metabolism [33]. In the current study, HDLC was significantly lower in the DM with and without DR groups than in the control group, suggesting that lower HDLC levels may have been associated with diabetic pathogenesis.

Given that adenosine triphosphate (ATP)-binding cassette protein A1 (ABCA1) contributes to the efflux of excessive cholesterol, which subsequently produces HDLC [34], serum HDLC levels may elevate with increased cholesterol loading, however, this only occurs if the efflux system functions appropriately. The cholesterol efflux system becomes impaired with constant lipid overloading through angiotensin II type 1 receptor signaling, as shown in obese mice generated by the constant intake of a high-fat diet [35]. Under abnormal metabolic conditions, cholesterol may be taken up and accumulated in the cells, which may not raise or may decrease extracellular lipid levels including HDLC levels.

**Fig 2:**
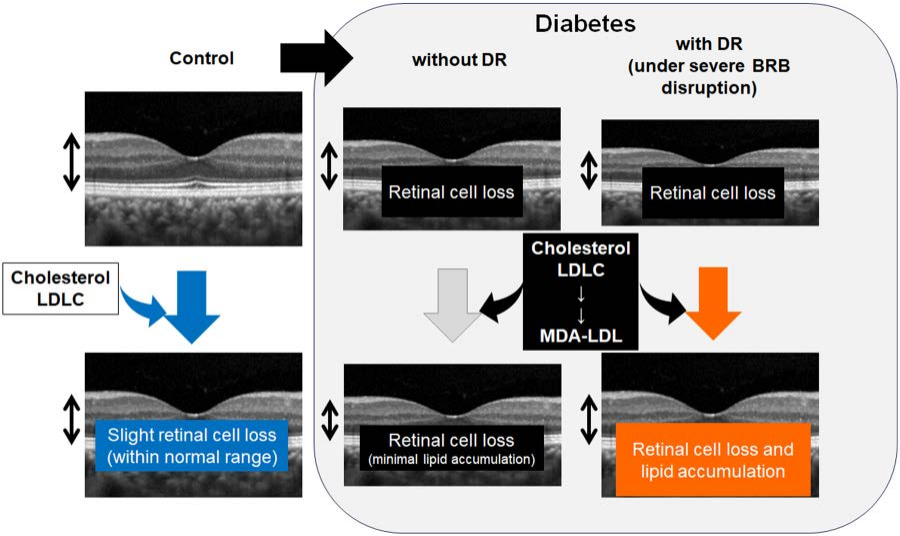
Hypothesis of the influence of lipids on retinal volume. In control and healthy eyes, high levels of serum cholesterol and low-density lipoprotein cholesterol (LDLC) may cause slight retinal cell loss to reduce the retinal volume, although the volume may remain within normal range. In eyes with diabetes, reduction in the retinal volume due to retinal cell loss is apparent. If the serum levels of lipids such as cholesterol and LDLC as well as malondialdehyde-modified low-density lipoprotein (MDA-LDL) which can be induced from LDLC are high, and the blood-retinal barrier (BRB) disruption is severe, the retinal volume may increase due to lipid accumulation; this may be one of the mechanisms of the current result that the retinal volume was positively correlated with serum lipid levels in the eyes with diabetic retinopathy (DR). In the eyes with diabetes with no DR, the BRB disruption may not be severe and the lipid accumulation in the retina may have been minimal.

The GCL volume was negatively correlated with TC and LDLC levels in the control group in the current study. In knockout (KO) mice lacking ABCA1, which is abundantly expressed in the GCL [36], cholesterol accumulates in the GCL, leading to RGC death, possibly due to mitochondrial dysfunction and oxidative stress [37]. Thus, the negative correlation observed between the GCL macular volume and TC and LDLC levels in the control group may represent retinal neural cell loss due to cholesterol accumulation, although it may be within the normal range (Fig.2).

However, the DM with DR group showed positive correlations between GCL volume and LDLC levels and between RNFL volume and TC, LDLC, and MDA-LDL levels, thus, the retinal volumes increased as the serum lipid levels increased, while no macular edema was observed. The correlation coefficient values showed opposite signs between the control and DM with DR groups, suggesting that different mechanisms may have determined macular retinal volume in these groups. Since inner retinal function is impaired in DM patients from the early phase, [10,11,13,14] and the GCL volume was reduced in the eyes of patients with DM before DR is diagnosed as shown in the current study and the previous report [21], the greater GCL macular volume in the DM with DR group may be pathogenic. The disruption of blood-retinal barrier (BRB) was most likely progressed in eyes with DR, thus, the lipids could have diffused through the disrupted BRB, and accumulated in the remaining retinal neural tissue, increasing the volume (Fig.2).

Fu et al. showed the accumulation of oxidized LDL in the retina of patients with DM through immunohistochemistry, particularly in the RNFL and GCL, and formed immunocomplexes with IgG. They observed the formation of immunocomplexes with immunoglobulin G (IgG), reporting that the complex became more toxic and finally induced apoptosis [38,39]. Accumulation of oxidized LDL can trigger the activation of the polyol and hexosamine pathways [40,41], and the oxidative stress [23,24,42], leading to tissue damage. The RNFL volume increased when MDA-LDL levels were elevated in the DM with DR group in the current study, suggesting that MDA-LDL may have diffused through the disrupted BRB, accumulated in the retinal layers composed by remaining neural cells, and increased the tissue volume, despite a potential reduction in the number of neurons due to DM.

Given that LDLC can produce MDA-LDL in circulation and target tissues, serum LDLC might have been also converted to MDA-LDL and accumulated in the neural tissue. Consequently, the NRL, which involved most of the neural retinal layers, was also positively correlated with serum TC, LDLC, as well as MDA-LDL in the DM with DR group. The result was consistent with our previous study in patients with DM without or with minimal DR (and without detectable diabetic macular edema), showing an association between increased serum LDLC and total layers of macular volume [43]. Considering that oxidized LDL and LDLC may have originated from the bloodstream, minimal fluid (not detectable in OCT images) and other serum components might have also contributed to the increased volume.

This study had limitations. First, it was performed with a relatively small sample size at a single institution, in a cross-sectional design. Second, this study was conducted in a clinical setting and did not involve pathological and molecular analyses of the retinal tissue. However, this study proposed potential new insights into the influence of lipid metabolism on DR, and the interpretations of macular retinal volumes in DR.

The current analyses of the associations between systemic lipid levels and the neural retinal volume at the macula showed opposite associations in the control and DR groups. The retinal volume may not necessarily represent the number of neurons, but may involve the additional pathological modifications such as accumulation of lipids in the retina particularly in the relatively advanced stage where BRB disruption has progressed and DR is visible, even in the absence of detectable macular edema. This study suggests the potential influence of lipids on DR pathogenesis, and emphasized the need for caution when assessing neurodegenerative changes in OCT images in clinical practice and future trials for novel therapeutic interventions. Further studies on the mechanisms of lipid metabolism in diabetic vascular and neural changes are required.

## Supporting information

S1 Data Raw data of the participants. (XLSX)
